# Trust and Compliance with COVID-19 Preventive Behaviors during the Pandemic

**DOI:** 10.3390/ijerph18052643

**Published:** 2021-03-05

**Authors:** Liat Ayalon

**Affiliations:** Gabi Weisfeld School of Social Work, Bar Ilan University, Ramat Gan 52900, Israel; liat.ayalon@biu.ac.il

**Keywords:** trust, compliance, policy measures, COVID-19, health behaviors, legitimacy, politics

## Abstract

This study examined the role of trust in lay people’s health behaviors related to the current pandemic. A total of 376 Israelis completed an online questionnaire during the second lockdown. A latent profile analysis was conducted to identify profiles of individuals based on their levels of trust in the various institutions and stakeholders examined in this study. A three-profile solution was deemed most appropriate. The largest profile (*N* = 178) was characterized by low levels of trust in the government, but high levels of trust in science and one’s primary care provider. Next, was the generally low trust profile (*N* = 108), characterized by low levels of trust directed towards all stakeholders and institutes. The third profile (*N* = 79) was characterized by high levels of trust. Results are discussed in relation to the important role of trust in determining people’s response to the current pandemic and the unique features of Israeli society.

## 1. Introduction

The term trust is defined as “the firm belief in the reliability, truth, ability or strength of someone or something.” The relationship between COVID-19 and trust deserves special attention for two major reasons. First, even seven months into the outbreak of the COVID-19 pandemic, we are still facing high levels of uncertainty regarding the nature of the disease, its lethality, spread and the best ways to reduce or prevent its impact [1]. As a result, different countries have responded in quite different ways to the same pandemic, and no gold standards have been established [2,3,4]. Second, current physical distancing guidelines provided to prevent the spread of the pandemic stand in clear contrast to our everyday lives and our nature as human beings and therefore, require profound adjustments and major changes in our behaviors [5]. Hence, on the one hand, there is a high degree of uncertainty concerning what constitute adequate measures to address the pandemic with different messages being voiced by the same individual over time, even in a matter of days or hours as well as by different individuals at the same time in the same country or in different countries. However, on the other hand, the measures required to address the pandemic are extensive and demand high levels of trust for people to deviate so substantially from their daily routines [1,6,7].

Under such circumstances, this study was set to examine the role of trust in lay people’s health behaviors related to the current pandemic. Theoretically, the issue of trust has been examined both in relation to interpersonal relations and in relation to institutions [8]. It has been argued that in contrast to trust in other people, trust in institutions is abstract as it does not rely on the actual encounter with the institutions. As such, the trustworthiness of institutions is determined by the level of trust developed as a result of encounters with the representatives of such institutions [9]. I examined trust in various national and international bodies currently responsible for the management of the pandemic, including the government, science, the World Health Organization, the health care system, one’s family physician, the traditional media and the new media. This represents a non-exhaustive list developed based on a review of existing literature. Below, I briefly demonstrate the issue of trust in relation to each of these entities. 

### 1.1. The Government

The present study was conducted in Israel. The Israeli government and governmental response to the pandemic have received much attention given the fact that the pandemic has occurred during a political deadlock, an unprecedented constitutional crisis, and a prime minister, facing a criminal trial, on the grounds of bribery, fraud, and breach of trust. The government’s reaction to the pandemic was portrayed as being disproportionate, both rhetorically and practically. At the same time, however, there were multiple errors of omission or commission of guidelines and policy measures, many of which, guided by political, rather than healthcare considerations [7]. 

In the most extreme period of lockdown imposed during the first wave of the pandemic, the public was instructed to stay within 100 m away from home, whereas during the second wave, a 1km restriction was imposed. Between the two waves, the prime minister, who has been holding multiple media appearances throughout the pandemic has instructed the public to go back to life and have fun. Hence, although the initial response and outcomes of the pandemic management in Israel have been portrayed as successful, the second wave of the pandemic had occurred quite early and resulted in a very large number of infected individuals, relative to the size of the population. This has been accompanied by a growing political upheaval accompanied by repeated ongoing demonstrations of Israeli citizens, who have been protesting on multiple grounds from the perceived illegitimacy of the Prime Minister to the financial crises brought by the restrictive measures implemented by the government or the sense of discrimination and unfair treatment of some segments of the population. Moreover, multiple political figures, including the Prime Minister, Benjamin Netanyahu, have been repeatedly violating and breaking the exact same orders they had issued to fight the pandemic. Thus, the contribution of leaders as role models [10] has been completely absent in current Israeli political scene.

As reported in past research, the acts (or lack of acts) taken by governments to protect their citizens have been highly associated with the level of trust in governments reported by these same citizens [11]. Research on the role of the government and COVID-19 has shown that at least in some countries, such as Hong Kong [3], against all odds, the government has managed to contain the pandemic adequately and gain the compliance of the citizens, despite limited trust in the government held by the public. In other countries, such as New Zealand or Norway [2], countries already characterized by high levels of trust, the government’s response to the pandemic has been described as adequate and the pandemic has been largely confined. In support of these findings, a study conducted in Germany has shown that trust in politics (and science) is associated with greater compliance with recommendations [1].

### 1.2. Science

In the face of the unknown, science plays an important role in characterizing the pandemic, its lethality and spread as well as ways of coping with it [12]. Early on, researchers and scientists have gathered forces to tackle the pandemic. These efforts, however, have occurred during an already contested period, when trust in science has been eroding among some political sectors [13]. Politics has been particularly influential in the public response to science around climate change as well as vaccination policies [14,15]. Whereas conservatives are more likely to question the science behind climate change, it is liberals, who are more likely to contest the science behind vaccines [16]. Under such circumstances, trust in science largely varies based on race, religion, political affiliation, social class and gender among other things [13]. Nonetheless, trust in science has played a prominent role in people’s engagement in health prevention behaviors during the current pandemic, as well as during past health atrocities [10]. 

### 1.3. The World Health Organization (WHO)

The WHO views its role as a leader on matters related to health, responsible for shaping the research agenda, synthesizing and disseminating relevant knowledge, building sustainable institutional capacity, articulating ethical and evidence-based policy options, and monitoring health status and trends [17]. These roles are essential in the face of a global unfamiliar threat, such as the COVID-19 pandemic. The COVID-19 was declared a pandemic by the WHO as early as March 11th, though it issued its first warning already in January. Since then, the Organization has developed multiple guidelines and information sheets concerning the pandemic. Nevertheless, criticism concerning the Organization has been voiced by several countries, including the United States and Australia. These countries have asked for the initiation of an independent review committee to assess the handling of the pandemic by the WHO as well as for restructuring the Organization [18]. Others have criticized the WHO for failing to allocate resources to address some of the most vulnerable populations, such as older adults [19]. As governments themselves have been skeptical about the WHO response and guidelines to the pandemic, it is likely that the public trust in this Organization, whose involvement has been portrayed as quite peripheral during the current global pandemic, has eroded as well. 

### 1.4. The Healthcare System and the Primary Physician

Early on, it has been made clear that the “starving and highly neglected” healthcare systems worldwide will not survive a full-blown pandemic, with millions of infected and dying people. In fact, the measures enacted by governments, worldwide, have been guided by the effort to save the healthcare system, in each country, rather than by a direct effort to protect their citizens [20]. This is evident by the fact that other impending risks such as air pollution, smoking, the global warming, obesity, or sedentary lifestyles have not received the same level of attention. While the former public health threats pose future risks, COVID-19 poses very imminent, immediate ones.

The healthcare system in Israel is considered a highly robust and trustworthy system but has suffered from extreme neglect over the years. Its hospitals are already over-crowded and fully occupied, even beyond full capacity as the ratios of nurses, physicians and hospital beds to citizens are low relative to other Organisation for Economic Co-operation and Development (OECD) countries [21,22]. Clearly, the management of a global pandemic when the entire healthcare system is already overcrowded and overburdened can be extremely challenging. Moreover, the minister of health in the country has violated the measures, he, himself had enacted to fight the virus. This likely has resulted in limited trust in the healthcare system in the country.

In a sharp contrast to the general healthcare system, which is constantly failing its customers, one’s relationships with his or her individual physician might still be characterized by high levels of trust [10]. In fact, past research has shown that patient-physician relations are of utmost importance for a variety of health behaviors, including preventive medical visits [23] and compliance with medication regimes [24].

### 1.5. The Media

The media can be divided into new (e.g., Twitter, Facebook) and old or traditional (e.g., television, newspapers). In the past two decades, there has been an ongoing shift from the reliance on the more traditional media to the new media, also known as social media. This transition to the new media is characterized by an influx of fake news and increasingly polarized societies. In the case of COVID-19, fake news is thought to be spreading at a faster pace than the pandemic itself [25]. It has been argued that the pandemic has brought with it an infodemic, characterized by an overload of inaccurate, misleading information [26]. This problem has been thought of as being severe enough to require the collaboration of the WHO with media platforms to better monitor and possibly mitigate the impact of fake news on the social media [27]. Nonetheless, the portrayal of inaccurate, superficial information is not limited to the new social media, as the traditional media platform also is thought to contribute to the spread of misinformation in the case of COVID-19 [4].

These global trends likely coincide with local events, as even prior to the pandemic, there have been high levels of skepticism towards mainstream news channels due to their reliance on sectorial and extranational news [28]. Moreover, over the past decade, there has been an ongoing erosion of the “free media,” which has been increasingly controlled and directly influenced by the Israeli Prime Minister, Benjamin Netanyahu and his collaborators [29]. In this climate, trust in both new and traditional media has likely been eroded.

### 1.6. The Present Study

During a global crisis, characterized by high levels of uncertainty and increasing demands for behavioral change in the face of the unknown, trust plays a major role. This study examined the relationship between compliance with health behaviors and trust during the second lockdown, imposed on Israel during September–October 2021. This study adds by examining the public trust in a breadth of possible stakeholders or institutions, taking a comprehensive approach to the issue. As trust was measured across a variety of possible stakeholders and institutions, this study aimed to identify public profiles of Israeli citizens distinguished based on degree of trust. The association of these profiles of trust with COVID-19 preventive behaviors and demographic characteristics was then examined.

## 2. Methods

The study was approved by the ethics committee of the PI’s university and all participants signed an informed consent. We relied on a convenience sample recruited via an online survey agency. Respondents received reimbursement for their time. Between 7th October and 15th October, when the state of Israel was under strict lockdown (1 km confinement), 376 people completed an online survey about their experiences during lockdown. Data are available upon request. Table 1 outlines the characteristics of the sample. The average age of the sample was 45.35 (18–71) and the majority were women with high school or first-degree education. The majority self-identified as traditional in terms of their religious beliefs.

### 2.1. Measures

#### Level of Trust

Respondents were asked: “please indicate on a scale of 0 to 100, how much do you trust any of the following institutes with the management of the Corona crisis.” The following institutes were subsequently presented and ranked by respondents: the government, science, primary care physician, healthcare system, the WHO, the traditional media and the new media.

COVID-19 preventive behaviors were assessed using 13 items, including: obtain COVID-19 health information, wash hands, use disinfectants, maintain good personal hygiene, wear a face mask, avoid traveling to areas infected by COVID-19, avoid public transportation, avoid sick people, avoid flights, avoid shaking hands, avoid eating out, avoid large gatherings, avoid work or school. These questions were ranked on a 1–4 scale, with 4 indicating greater frequency, with a higher overall score representing greater compliance. The reliability of overall compliance with COVID-19 preventive behaviors was Cronbach’s alpha of 0.81. 

Demographic variables including age, sex, and education (1–5, less than high school education-second degree or higher) were gathered based on self-report.

### 2.2. Analysis

Descriptive statistics and correlations were obtained via R [30]. Latent profile analysis was calculated using M-Plus [31]. The approach assumes that there are unobserved groups of people that can be classified based on their response to each of the seven trust indicators. Hence, the assumption is that there is a pattern in the response participants provide, which can be recognized using latent profile analysis. It is assumed that unobserved variability can be explained through the classification of the variability among observed variables (e.g., the trust indicators) [32]. Latent profile analysis accounts for the measurement error, relies on a probability-based approach, and provides a statistical test for the appropriate number of profiles.

The seven trust indicators were entered into a mixture modeling procedure. Latent profile analysis provides a flexible approach to determine the number of potential profiles to model observed variables within the profiles [32]. The model with the lowest number of profiles and best fit indices is selected. Fit indices are determined based on the Akaiake Information Criteria (AIC) and the Bayesian Information Criteria (BIC). Lower values indicate better fitting models. The Lo–Mendell–Rubin adjusted likelihood ratio test provides a statistical response as to whether or not an additional profile improves the fit of the model [33]. A significant *p*-value suggests that the model provides a better fit to the data compared with a model with one less profile. Entropy scores indicate the degree to which profiles are differentiated from one another. The closer the entropy score is to 1, the better the prediction is. The outcome of the analysis is the assignment of each individual to a profile. A good fitting model has a high probability of classifying each case to only one profile.

The size of each profile also can be used to determine the overall number of profiles as very small profiles represent spurious results. To ensure the stability of the models, different sets of starting values based on the local maximum in the iteration process were specified [34].

Once a profile solution was established based on the fit indices described above, it was examined against the descriptive characteristics of the sample as well as against the COVID-19 preventive behaviors overall score.

## 3. Results

As can be seen in Table 2, there were significant correlations between most trust variables ranging between 0.21 (primary care physician-government) and 0.62 (primary care physician-science). The lowest level of trust was reported regarding the government and the new social media, whereas the highest level of trust was reported with regard to the primary care physician followed by the healthcare system. Table S1 in the Supplementary File demonstrates the fit indices of the latent profile solutions examined. I started from a single-profile solution and moved until we reached a four-profile solution. The three- profile solution was selected as it has the best fit indices, including low AIC, BIC and the highest entropy score, indicating highly distinguished profiles. Moreover, the Lo–Mendell–Rubin adjusted likelihood ratio test indicated that a four-profile solution was not significantly better than a three-profile solution.

Table 3 describes the three-profile solutions in relation to its seven trust indicators. Respondents were classified into one of three profiles based on the level of trust they reported in relation to each of the indicators. As can be seen, profile 1, is characterized by general low levels of trust. Respondents’ trust in all institutes was relatively low and with the exception of one item, trust was significantly lower than the other profiles. This profile is distinguished from the other two profiles on all indicators, with one exception, namely trust in government, which was not significantly different from that reported by those classified into profile 2. Profile 2, named “limited trust in government,” is characterized by high levels of trust in science and one’s primary care physician, which are comparable to that reported by the high trust profile, 3, medium levels of trust in the healthcare system and low levels of trust in the government, which, as already noted, is not distinguishable from that reported by the low trust profile. The final profile was characterized by high levels of trust on all seven indicators. Thus, respondents classified into this profile reported higher levels of trust on all seven indicators, compared with those classified to the other profiles.

Next, the three- profile solution was examined against demographic characteristics and overall compliance with COVID-19 preventive behaviors. These are reported in Table 4. The generally low trust profile was significantly younger and was less likely to engage in COVID-19 related health behaviors compared with the other two profiles.

## 4. Discussion

This study examined whether individuals can be characterized according to the level of trust they report regarding major stakeholders or institutes of potential relevance to the implementation and enforcement of the current health related COVID-19 guidelines. Our findings point to the presence of three distinct profiles of individuals, characterized based on their levels of trust in various institutes and stakeholders of relevance as well as to the importance of trust in establishing people’s compliance with current COVID-19 guidelines.

The highest levels of trust were reported regarding primary care physicians and science. This is reassuring given the important role assigned to the primary care provider in guiding patients through their health regimes [24]. People’s confidence in science also was high. This too is extremely important given the role confidence in science is likely to play in people’s willingness to vaccinate once a COVID-19 vaccine becomes relevant [35]. In contrast, Israelis expressed low levels of confidence in their government and in the media, especially the new media. These findings are likely context dependent. Israeli society has become extremely polarized and volatile during the current pandemic, with resentment being directed mainly towards the government and occasionally towards the media, but almost no direct criticism being directed towards the declining healthcare system, as the direct responsibility to the situation of the healthcare system has been attributed to the government, rather than to the system itself [36]. In fact, like other places around the world [37], Israelis have been extremely grateful and appreciative of the healthcare workforce.

A three-profile solution emerged as the most adequate solution to explain the variability in people’s responses regarding their level of trust in the various stakeholders and institutes examined in this study. The first profile, which was the second largest, was characterized by general low levels of trust in all stakeholders examined in this study. Individuals classified into this profile were significantly younger than those in the other two profiles. Past research has shown that younger people are disproportionally stressed, anxious and lonely during the pandemic [38]. Possibly their limited trust in various institutions contributes to their emotional state during the current pandemic.

The profile characterized as having general low levels of trust also was the least likely to comply with COVID-19 guidelines in this study. Hence, consistent with past research [39,40], our findings point to a potential risk for non-compliance with current guidelines associated with low levels of trust. The present findings may also explain some of the reports concerning younger people’s disregard to COVID-19 physical distancing guidelines [41]. Moreover, according to the Israeli Ministry of Health, younger people are disproportionately infected by COVID-19. Hence, this study adds by suggesting that this might be due to limited compliance with current guidelines associate with low levels of trust.

The largest profile in this study consisted of individuals who reported very low trust in the government, comparable to those reported by the generally limited trust profile, but high levels of trust in science and one’s primary care physician at comparable levels to those reported by the high trust profile. Low levels of trust in the government can be attributed to the highly polarized and contested political discourse in the country [7]. Nonetheless, although the largest profile was composed of individuals who had low trust in the government, they still held high trust in the other stakeholders and institutes. In its characteristics and approach to COVID-19 guidelines, this group was not distinguished from the high trust group. Thus, our findings show that the political instability in the country and the low trust in the Israeli government have limited impact on respondents’ COVID-19 related health behaviors.

Of note is the fact that the sample was not distinguished regarding its religiosity. This is unexpected given the fact that most infected cases in Israel have been ultra-orthodox [42]. Although a large portion of our sample self-identified as religious, we did not reach the ultra-orthodox community and thus, our study is limited in that regard. Another limitation of the present study concerns its cross-sectional nature, which does not allow for inferences about cause and effect. Nonetheless, this study provides an innovative approach to the understanding of trust in relation to the COVID-19 outbreak. Our findings allude to three distinct Israeli groups that can be distinguished based on their level of trust in various institutions and stakeholders. The findings also suggest that younger people, characterized by low levels of trust, are those least likely to engage in COVID-19 preventive behaviors and, thus, require the most attention and explanation regarding the importance of following current guidelines.

## Figures and Tables

**Table 1:** Demographic characteristics of the sample (*N* = 376) (Note. M and SD are used to represent mean and standard deviation, respectively. F represents frequency).

Variable	M/F (%)	SD
Age	45.35	14.28
Women	207 (55.3%)	
Education		
Less than elementary education	0	
Elementary education	4 (1%)	
High school education	146 (39.9%)	
First degree	146 (39.9%)	
Second degree and higher	70 (19.1%)	
Religiosity		
Secular	112 (31.9%)	
Traditional	140 (39.9%)	
Religious	99(28.2%)	

**Table 2:** Means, standard deviations, and correlations of trust indicators (*N* = 376).

Variable	*M*	*SD*	1	2	3	4	5	6
1. Government	23.77	27.63						
2. Science	64.47	25.98	0.26 **					
3. Primary care physician	57.24	27.07	0.21 **	0.62 **				
4. Health care system	50.13	28.63	0.49 **	0.53 **	0.58 **			
5. The World Health Organization	47.77	29.97	0.34 **	0.51 **	0.37 **	0.59 **		
6. Traditional media	31.29	26.42	0.26 **	0.35 **	0.43 **	0.40 **	0.43 **	
7. New media	22.53	22.78	0.33 **	0.19 **	0.22 **	0.25 **	0.29 **	0.54 **

Scores range between 0 and 100, with a higher score indicating greater trust. M and SD are used to represent mean and standard deviation, respectively. ** indicates *p* < 0.01.

**Table 3:** Trust indicators of the three-profile solution (*N* = 376).

	Profile 1-Limited Trust in General (*N* = 108)	Profile 2-Limited Trust Primarily in the Government (*N* = 178)	Profile 3-High Trust (*N* = 79)	F(df)
1. Government	9.12 ^a^ (12.77)	11.47 ^a^ (12.68)	66.37 (17.93)	530.17 ** (372.2)
2. Science	35.90 (22.53)	75.06 ^a^ (16.55)	77.73 ^a^ (17.21)	178.50 ** (372.2)
3. Primary care physician	28.22 (18.18)	68.63 ^a^ (20.46)	77.17 ^a^ (20.62)	161.64 ** (372.2)
4. Healthcare system	20.12 (15.06)	56.70 (23.77)	73.83 (17.43)	190.83 ** (372.2)
5. WHO	22.31 (21.03)	53.92 (26.16)	66.07 (26.36)	86.29 ** (372.2)
6. Traditional medial	13.92 (18.15)	33.89 (24.83)	46.89 (26.43)	50.13 ** (372.2)
7. New media	13.01 (17.62)	20.39 (20.35)	38.19 (25.10)	37.25 ** (372.2)

^a^ denotes non-significant Bonferroni differences between profiles. Otherwise, profiles are significantly different from each other at ** *p* < 0.01.

**Table 4:** Demographic characteristics and health behaviors and the three-profile solution.

	Profile 1-Limited Trust in General (*N* = 108)		Profile 2-Limited Trust Primarily in the Government (*N* = 178)		Profile 3-High Trust (*N* = 79)		
Variable	M/F (%)	SD	M/F (%)	SD	M/F (%)	SD	F(df)/χ^2^
1. Age	39.39	14.27	47.03 ^a^	14.05	47.22 ^a^	13.03	11.69 ** (371.2)
2. Sex	58 (54.2%)		106 (59.6%)		43 (48.3%)		3.11 (2)
3. Education	3.78	0.74	3.74	0.78	3.78	0.77	0.13 (371.2)
4. Secular	26 (36.6%)		38 (37.3%)		14 (25.9%)		3.11 (2)
Traditional	25 (35.2%)		44 (43.1%)		19 (35.2%)		
Religious	20 (28.2%)		20 (17.6%)		21 (38.9%)		
5. Health behaviors	2.90	0.69	3.20 ^a^	0.50	3.27 ^a^	0.49	12.51 ** (372.2)

^a^ denotes non-significant Bonferroni differences between profiles. Otherwise, profiles are significantly different from each other at ** *p* < 0.01.

## Data Availability

Data are available upon request.

## Supplementary Materials

The following are available online at https://www.mdpi.com/1660-4601/18/5/2643/s1, Table S1: Latent trust profiles fit indices.
