# Supplementary material for: Trust and Compliance with COVID-19 Preventive Behaviors during the Pandemic
Source: Int J Environ Res Public Health. 2021 Mar 5;18(5):2643. doi: 10.3390/ijerph18052643 (PMC7967340; doi:10.3390/ijerph18052643)
Supplement: Supplementary file 1 [file ijerph-18-02643-s001.pdf]

Table S1. Latent trust profiles fit indices

	AIC	BIC	Loglikelihood	Entropy	Lo-Mendell-Rubin Adjusted Lrt Test	*p*
1	24808.26	24863.28	-12390.13			
2	24211.89	24298.34	-12390.13	0.809	599.729	0.002
**3**	**24015.90**	**24133.79**	**-12803.94**	**0.854**	**207.61**	**0.005**
4	23931.44	24080.77	-11609.966	0.806	98.38	0.056

Akaiake Information Criteria (AIC); Bayesian Information Criteria (BIC)
